# 7-Tesla sodium magnetic resonance imaging of the inner ears in unilateral Ménière’s disease and endolymphatic hydrops: an exploratory study

**DOI:** 10.1186/s12880-025-01986-6

**Published:** 2025-11-11

**Authors:** Steve Connor, Peter Lally, Irumee Pai, Haneefah Brnawi, Philip Touska, Sebastien Ourselin, Joseph V. Hajnal

**Affiliations:** 1 School of Biomedical Engineering and Imaging Sciences, King’s College London, London, SE1 7EH UK; 2 Department of Neuroradiology, King’s College Hospital, London, SE5 9RS UK; 3 Department of Radiology, Guy’s Hospital and St Thomas’ Hospital, London, SE1 9RT UK; 4 Department of Bioengineering, Imperial College London, London, W12 0BZ UK; 5 Department of Ear, Nose and Throat Surgery, Guy’s and St Thomas’ Hospital, London, SE1 9RT UK

**Keywords:** Magnetic resonance imaging, Meniere’s disease, Ultra-high-field MRI, Endolymphatic hydrops, Sodium

## Abstract

**Background:**

Whilst delayed post-gadolinium MRI has led to a shift in the diagnostic paradigm of Meniere’s Disease (MD), there remains a strong desire to develop a non-contrast enhanced MRI technique to detect and monitor MD. The endolymphatic space (ES) undergoes hydropic expansion in Ménière’s Disease (MD) and the concentration of sodium ions in the endolymph is at least 10 times lower than that in the perilymph. It was hypothesised that the lower sodium (^23^Na) concentration in the endolymph relative to the surrounding perilymph would result in a differential reduction in ^23^Na-MRI signal in inner ears with endolymphatic hydrops (EH). This proof of principle study explored the feasibility of 7-Tesla (7T) ^23^Na-MRI to lateralise EH ears in unilateral MD.

**Methods:**

In this prospective study, 7T ^23^Na-MRI was performed in participants with both unilateral definite MD and severe vestibulo-cochlear EH on a delayed post-gadolinium real inversion recovery sequence. Two blinded independent observers qualitatively graded the visibility and anatomical compatibility of inner ear ^23^Na MRI signal intensity (NaSI), before and after registering to 3D T2-weighted (T2w) MRI and determined the certainty of EH laterality. The internal auditory meatus (IAM), cochlea and vestibule were segmented using 3D Slicer and NaSI was quantified. Inner ear median NaSI were scaled to the adjacent IAM median NaSI and compared between the two ears.

**Results:**

In 4 unilateral MD participants (mean age 60.3 years, 2 men), both observers correctly predicted EH laterality in 1/4 before and 3/4 participants after fusion to 3D T2w MRI. There was no incorrect lateralisation of EH by either observer, either before or after registration and fusion. In the 3 participants correctly lateralised, quantitative analysis revealed the median inner ear NaSI scaled to the ipsilateral IAM was 1.2–2.8 times higher in the normal cochlea and 1.9–2.9 times higher in the vestibule, compared to the EH ear. Intraclass correlation coefficient for inner ear median NaSI was 0.70.

**Conclusion:**

This exploratory study revealed the potential for severe EH to be qualitatively and quantitatively lateralised with 7T ^23^Na MRI in patients with unilateral definite MD.

**Trial registration:**

NCT04370366; registered 29/4/20.

## Background

The inner ear fluid comprises a central endolymphatic compartment which is separated by membranes from the surrounding larger perilymphatic compartment. The principle endolymphatic structures are the saccule and utricle of the vestibule, and the scala media of the cochlea. Ménière’s disease (MD) is an inner ear condition with a typical clinical presentation of episodic vertigo, low- to mid-frequency hearing loss and fluctuating aural symptoms and has a prevalence of up to 513/100,000 [1]. The pathological hallmark of MD is endolymphatic hydrops (EH) which represents an expansion of the endolymphatic space (ES) into the surrounding perilymphatic space (PS) [2]. Temporal bone studies demonstrate EH in 98.8% of patients with clinically suspected MD [3].

Differential permeability of the endolymph and perilymph to gadolinium-based contrast agents (GBCA) allows the enhancing PS to be distinguished from the non-enhancing ES on delayed post-GBCA MRI three-dimensional inversion recovery (3D-IR) sequences. 3-dimensional inversion recovery (3D-IR) sequences enable the detection of low concentrations of gadolinium in the perilymph with high resolution depiction of the inner ear compartments on delayed post-GBCA MRI. Following its initial description in 2007 [4], MRI has been increasingly utilised worldwide for the demonstration of EH. Meta-analysis has indicated a high sensitivity (87%) and specificity (91%) for the diagnosis of MD with optimal combinations of MRI descriptors [5]. The increasing role of MRI in hydropic ear disease, has led to proposals for imaging-based rather than clinical terminology [6] and the emergence of MRI features in diagnostic guidelines for MD [7].

Intravenous GBCAs have an excellent safety record but they are not exempt from safety considerations. A causative relationship between less stable linear chelate GBCAs and nephrogenic systemic fibrosis is described in patients with severe renal insufficiency. In addition, there is evidence of gadolinium deposition in the human brain after multiple administrations, although this is reduced with macrocyclic agents [8, 9]. Therefore, it would be beneficial to develop a non-contrast enhanced MRI technique to detect and monitor MD [10]. Whilst there are some reports of EH being demonstrated with non-contrast high resolution T2 weighted (T2w) [10] and 3D Fluid Attenuated Inversion Recovery (3D-FLAIR) sequences [11], these have not been widely reproduced.

The ES and PS have distinctive ionic compositions which are maintained by ion channels and transport mechanisms [12, 13]. The maintenance of ion haemostasis is required for hair cell function with endolymph surrounding the stereocilia and perilymph bathing the hair cell bodies. Hair cells depolarize with motion, so allowing the transduction of sound and head acceleration into the nerve impulses necessary for normal hearing and balance. One notable feature of the ionic environment is that the sodium (^23^Na) concentration in the ES (1.3 mM) is considerably lower than that of the PS (141–148 mM) [14]. It may be hypothesised that this differential biodistribution of ^23^Na concentration could be probed with ^23^Na MRI, and that the expanded ES in patients with MD would result in lower ^23^Na MRI signal relative to the normal ear. ^23^Na MRI signal is limited by the low natural abundance, rapid quadrupolar relaxation and lower gyromagnetic ratio of ^23^Na [15], although this may be partly mitigated by imaging at ultra-high field strength. Validation of ^23^Na MRI outside the brain, kidney and cartilage is limited, and imaging of the inner ears is particularly challenging due to magnetic susceptibility gradients at bone and air interfaces, small anatomical structures and suboptimal coil sensitivity for deep skull base structures.

We aimed to explore whether a ^23^ Na 7 Tesla (7T) MRI sequence could distinguish pathological ears by detecting asymmetrically reduced ^23^ Na signal in patients with unilateral MD and EH.

## Method

### Participants

All methods in this prospective study were carried out in accordance with relevant guidelines and regulations and the experimental protocol was approved by the institutional ethical committee (Health Research Authority. Southwest Frenchay Research Ethics Committee. Initial approval 16/6/20. Amendment approval 15/11/22. IRAS ID: 259867, Rec Reference: 20/SW/0085). Informed consent was obtained from all subjects.

The Picture Archiving and Communication System (Sectra AB, Sweden) was interrogated for consecutive patients undergoing clinical delayed post-GBCA inversion recovery (IR) MRI between December 2017 and October 2022. These patients had presented with symptoms of EH including episodic vertigo, sudden-onset or fluctuating SNHL, aural fullness and tinnitus. The contemporary clinical and audiometric data was reviewed by two observers (SC, IP) who were blinded to imaging findings, and patients were classified according to the current 2015 Barany Society criteria [16]. Clinical data was recorded in the six months and audiometry in the twelve months prior to the MRI study.

The delayed post-GBCA IR sequences were independently analysed by two radiologists (PT, SC), with 7- and 25-years of subspecialty head and neck radiology experience. The radiologists rated the images according to the Nakashima criteria [17] whilst blinded to clinical diagnosis. Participants were eligible for the study when both observers recorded unilateral “definite MD” on clinical review and both radiologists recorded unilateral severe (Grade 2 Nakashima) vestibular and cochlear EH on imaging analysis. A priori exclusion criteria were inadequate contemporary clinical details for diagnostic classification, clinical or radiological suspicion of secondary hydrops, previous inner ear operations, and technically inadequate or degraded MR imaging. The first four eligible participants, determined by the date of their initial diagnostic MRI scan, were invited to take part in the research 7 tesla (7T) ^23^Na MRI study 7-tesla (^23Na) MRI study in accordance with the study protocol.

### ^23^Na MRI sequence and reconstruction development

^23^Na MRI of the inner ears was refined on a 7T Magnetom® Terra scanner, (Siemens Healthineers, Erlangen) using a 1Tx/32Rx ^23^Na head coil (Rapid Biomedical, Rimpar) through iterative phantom and healthy volunteer (HV) imaging. Initial HV sequences were performed with acquisition times of 40–55 minutes to evaluate parameter changes, which were applied to an approximately 30 minutes sequence that could be tolerated by participants. A 3D Cones *k*-space trajectory was utilised which allowed for very short echo times and repeat time (TR) of ^23^Na MRI, whilst providing increased signal to noise (SNR) efficiency and good motion properties [18]. A balanced steady-state free precession (bSSFP) sequence was employed [19] since it demonstrated greater SNR on phantom and HV studies compared to a fast low angle shot (FLASH) sequence. A disadvantage of the bSSFP sequence is its sensitivity to static field (B_0_) inhomogeneity in the region of the inner ears due to local air, bone and fluid interfaces, and this resulted in banding artefact which was exacerbated by the ultra-high field strength. Therefore, phase cycling was used to enable systematic displacement of band locations and reduction in band conspicuity through image summation [20]. A 3 mm voxel size provided suboptimal delineation of the inner ears, and a 2 mm voxel size was considered to provide an appropriate balance of SNR and spatial resolution. Blurring of the images was reduced with a 2 ms readout compared to 4 ms and 10 ms readouts. The TR remained as required for the longer readout of 10 ms. A further reduction in TR would have precluded the application of a high enough flip angle for efficient fluid imaging whilst remaining within specific absorption rate (SAR) limits.

The final research 7T ^23^Na MRI study comprised a bSSFP phased cycled sequence and a 3D cones trajectory: 1 ms radiofrequency pulse width, 2x2x2 mm resolution, 240 mm^3^ field of view, flip angle 25°, TR 14.26/TE 1.06 ms, 2 ms readout and 5 phase cycles (72° increments) with time per phase cycle 6.24 min. A complex summation of the five phased cycled datasets, each adjusted by its own linear phase cycling increment ($\Delta \phi$), resulted in three signal components (F_0_, F_1_, and F_-1_) [21]. This applied the following expression:

$$data({F_n}) = \sum\limits_{\Delta {\rm{\varphi }}} {dat{a_{\Delta {\rm{\varphi }}}}} {e^{ - i\Delta {\rm{\varphi }}n}}$$

where the linear phase cycling increment, $\Delta \phi$, is in radians and n is the order of the F-state [19].

Each data set was reconstructed via nonuniform-FFT (conjugate-gradient SENSE) [22], and a composite image combined all three F-state components via root sum of squares [19]. The images were reconstructed with a frequency offset sweep set at 20 Hz steps from −100 to 200 Hz to correct for local susceptibility effects and were saved as 4 D stacks [X Y Z Hz offset] in NIfTI format.

### Clinical delayed post-gadolinium 3D-IR MRI sequences

Delayed post-gadolinium 3D-IR MRI sequences and three dimensional (3D) T2w structural imaging of the inner ears and been previously acquired according to routine clinical scanning protocols. The delayed post-gadolinium 3D-IR MRI sequences were used to assess participant eligibility whilst 3D T2w structural imaging were used for subsequent image fusion with ^23^Na MRI. Clinical MRI was performed on a 3T Magnetom® Skyra (Siemens Healthineers, Erlangen) scanner with a 64-channel head coil. 3D-IR sequences with phase corrected real reconstruction (3D real-IR) were acquired 4 hours after intravenous administration of gadoterate meglumine (0.2 mmol/kg) according to the institutional clinical protocol. 3D T2w imaging to delineate the inner ear anatomy was performed with a T2w sampling perfection with application optimised contrasts using different flip angle evolution (T2w SPACE). Siemens product sequences were used with parameters tabulated in Table 1.

**Table 1:** MRI parameters for clinical imaging of the inner ears

	3D real-IR	3D real-IR	T2-SPACE
Repetition time	6000 ms	15130 ms	1000 ms
Echo time	180 ms	550 ms	125 ms
Inversion time	2000 ms	2700 ms	NA
Number of excitations	1	1	2
Refocusing flip angle	180° (constant)	130° (constant)	100°
Pixel band width	220	435	255
Echo train length	27	267	52
Pixel spacing	0.7 mm	0.66 mm	0.31 mm
Slice thickness	0.7 mm	0.6 mm	0.3 mm
Matrix size	256 × 240	320 × 270	262 × 512
Field of view	190 × 178 mm	210 × 177 mm	80 × 160 mm
Acquisition time	13.38 min	11.21 min	6.38 min

### Registration and qualitative analysis

**Fig. 1:**
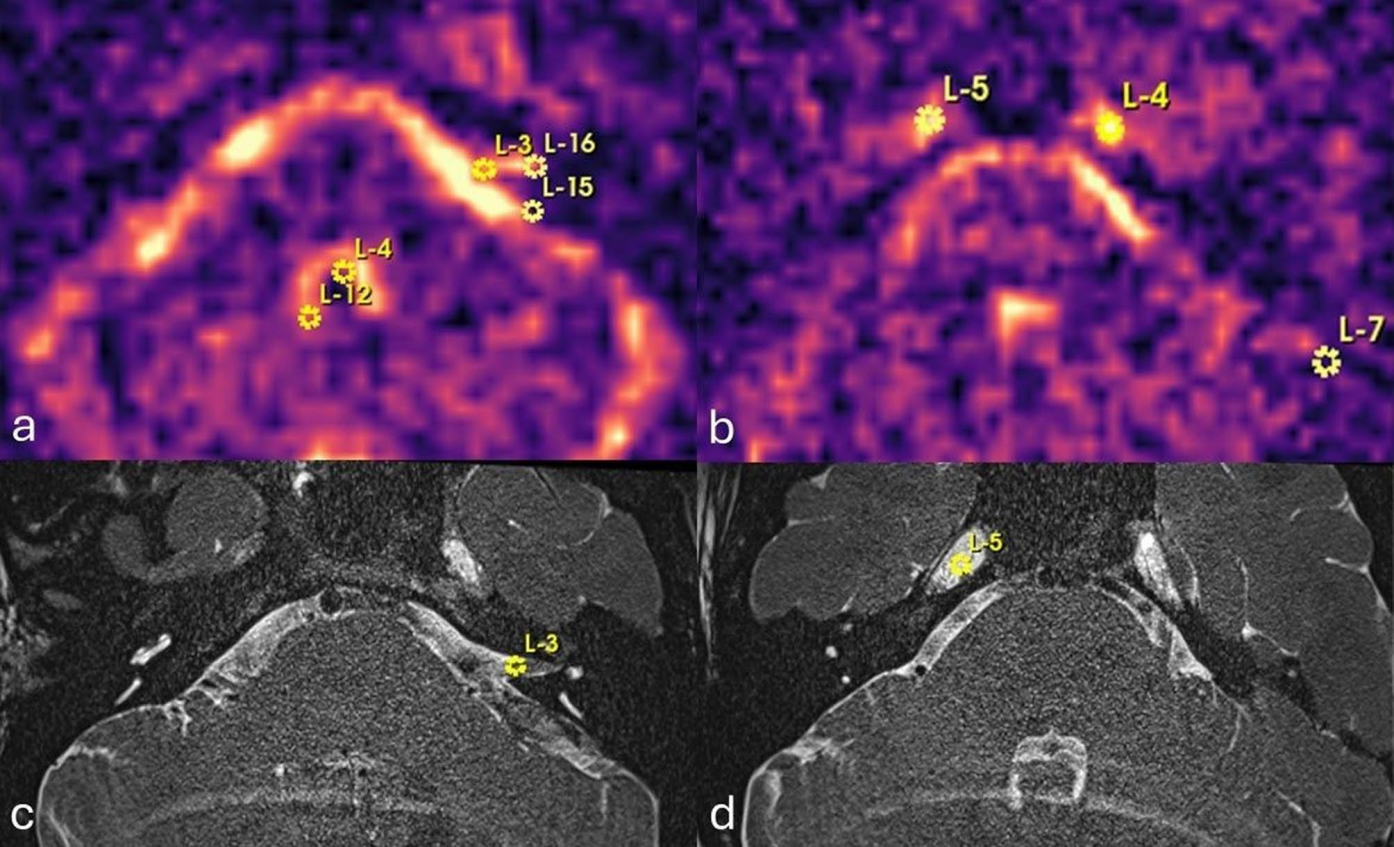
^23^Na MRI axial images demonstrating landmark placement for registration. These are placed in the (**a**) fourth ventricle (L-4, L-12), medial cerebello-pontine angle cistern (L-3, L-15) and internal auditory meatus (L-16) and (**b**) lateral cerebello-pontine angle cistern (L-7) and Meckel’s caves (L-4, L-5). Selected corresponding points are shown in (**c**) and (**d**) on the structural T2-SPACE sequences

The reconstructed combined F state ^23^Na MR images were viewed across the range of off-resonance frequencies using ImageJ [23]. The ^23^Na MR image quality and artefact (distortion, susceptibility and ghosting) was evaluated according to a Likert scale (Table 2) [24]. The optimal frequency offset for the depiction of each inner ear with minimal blurring was then selected. All ^23^Na MRI images grey scale imaging may be accessed in the supplementary data. Two observers (SC, IP) independently registered the ^23^Na MR images to the 3T T2w SPACE sequence for each ear using 3D-Slicer 5.6.2 [25] landmark-based registration, using 9–16 control points (Fig. 1). There was a priori agreement to use anatomical reference points in midline, paramidline and lateral posterior fossa structures at the interfaces of bone-cerebrospinal fluid and brain-cerebrospinal fluid structures, but the inner ear structures were not used since this was the target of analysis. The T2w SPACE sequence was chosen as the fixed volume and the ^23^Na MRI as the moving volume in thin-plate spline mode.

**Table 2:** Grading scales for image quality, artefact, signal visibility and anatomical compatibility

	Image quality		Artefact		Visibility		Anatomical compatibility
0	Non-diagnostic	0	Severe effect	0	Not visible	0	Structure not visible
1	Suboptimal	1	Moderate effect	1	Decreased signal relative to adjacent structure*	1	Major concern/minority ( < 50%) overlap**
2	Good	2	Minimal effect	2	Similar signal to adjacent structure*	2	Minor concern/major ( > 50%) overlap**
3	Excellent	3	No artefact	3	Increased signal relative to adjacent structure*	3	Entirely concordant

* *Adjacent structure* is the cerebello-pontine angle cistern for internal auditory meatus signal and the internal auditory meatus for inner ear (cochlea and vestibule) signal

** This may be because signal extends outside of the anatomical structure or only overlaps part of the anatomical structure

**Fig. 2:**
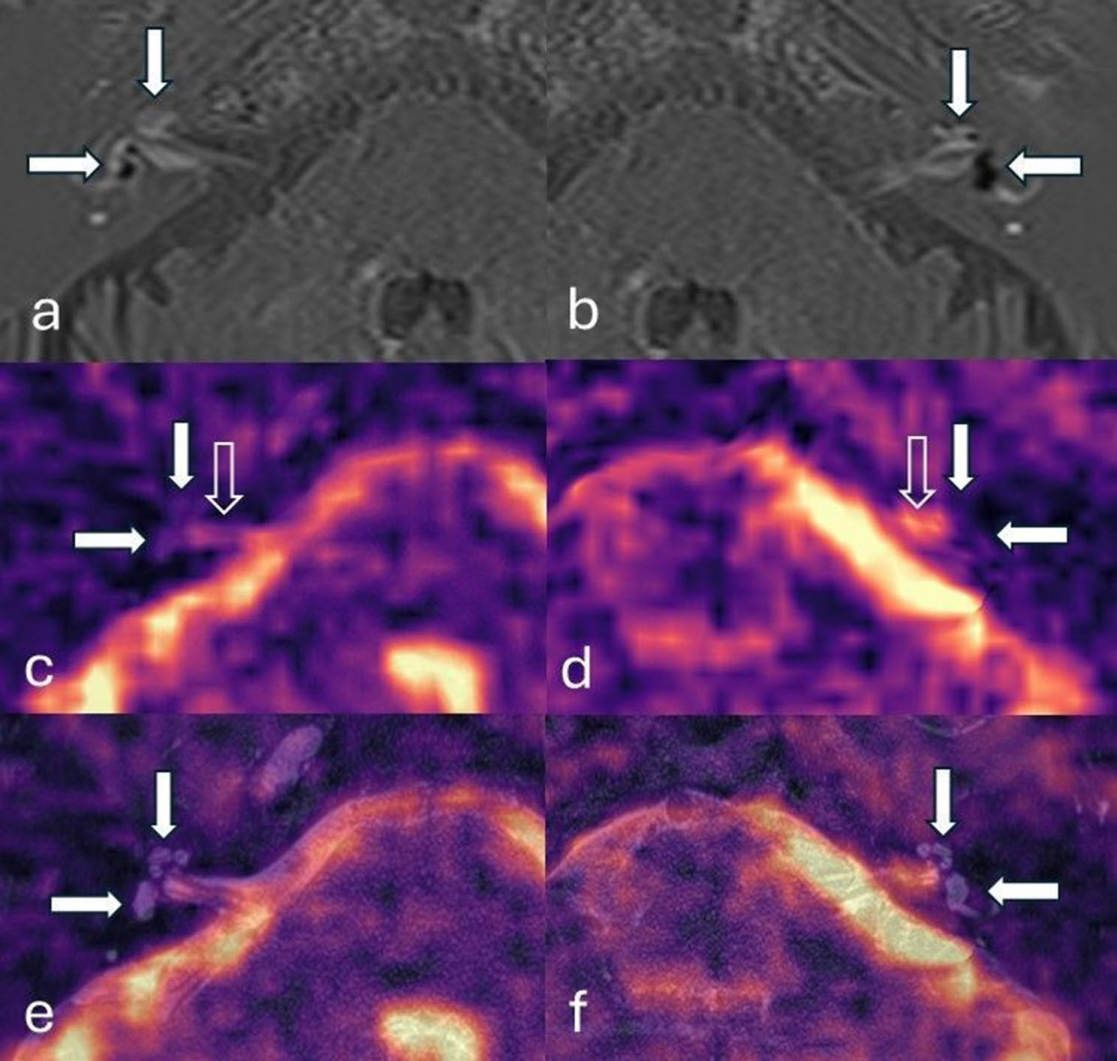
Delayed post-gadolinium 3D inversion-recovery (**a,b**), unfused ^23^Na MRI images (**c,d**) and ^23^Na MRI images registered/fused to ^23^Na MRI- T2-SPACE images (**e,f**). Participant C with left sided severe vestibulo-cochlear endolymphatic hydrops and good quality ^23^Na MRI images. The endolymphatic hydrops was correctly lateralised by observer 1 on ^23^Na MRI images both before and after registration/fusion to structural imaging. The cochlea (vertical filled arrow) and vestibule (horizontal filled arrow) are indicated. Delayed post gadolinium 3D inversion-recovery sequence demonstrates the enhancing perilymphatic space with small non-enhancing endolymphatic structures in the normal right ear (**a**) whilst there are dilated endolymphatic structures in the pathological left ear with endolymphatic hydrops (**b**). The enhancing left vestibular perilymph is completely replaced by the enlarged endolymphatic structures. Axial ^23^Na MRI images and registered/fused ^23^Na MRI- T2-SPACE images of the right ear (**c** and **e**) and left ear (**d** and **f**). The cochlea and vestibule are indicated on the registered/fused ^23^Na MRI- T2-SPACE images (**e, f**) and their expected locations are indicated on ^23^Na MRI images (**c, d**). The normal right ear (**a** and **c**) demonstrates ^23^Na MRI grade 1 signal relative to the adjacent internal auditory meatus (vertical open arrow), but with anatomical compatibility scored as grade 3 (entirely concordant). The pathological left ear with endolymphatic hydrops (**b** and **d**) did not demonstrate any visible ^23^Na MRI signal (grade 0) in the line of the inner ear structures on the registered/fused ^23^Na MRI- T2-SPACE images (**d**) despite clear visibility of signal within the internal auditory meatus (vertical open arrow). The ^23^Na MRI images grey scale imaging may be accessed in the supplementary data

**Fig. 3:**
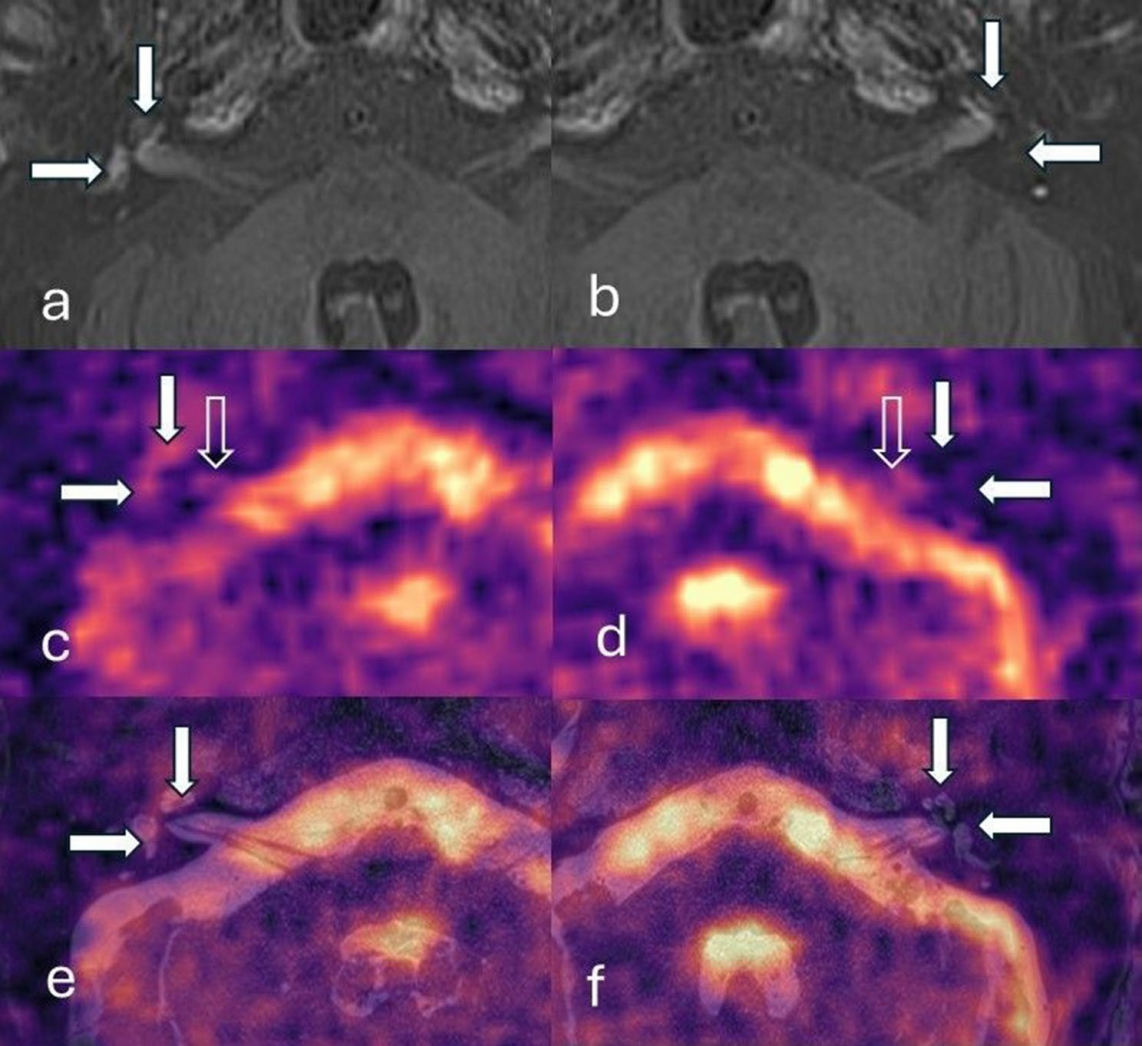
Delayed post-gadolinium 3D inversion-recovery (**a,b**), unfused ^23^Na MRI images (**c,d**) and ^23^Na MRI images registered/fused to ^23^Na MRI- T2-SPACE images (**e,f**). Participant a with left sided severe vestibulo-cochlear endolymphatic hydrops and suboptimal quality ^23^Na MRI images. The endolymphatic hydrops was only correctly lateralised by observer 1 on ^23^Na MRI images after it was registered and fused to structural imaging. The cochlea (vertical filled arrow) and vestibule (horizontal filled arrow) are indicated. Delayed post gadolinium 3D inversion-recovery sequence demonstrates the enhancing perilymphatic space with small non-enhancing endolymphatic structures in the normal right ear (**a**) whilst there are dilated endolymphatic structures in the pathological left ear with endolymphatic hydrops (**b**). Axial ^23^Na MRI images and registered/fused ^23^Na MRI- T2-SPACE images through the right ear (**c** and **e**) and left ear (**d** and **f**). The cochlea (vertical filled arrow) and vestibule (horizontal filled arrow) are indicated on the registered/fused ^23^Na MRI- T2-SPACE images (**e, f**) and their expected locations are indicated on ^23^Na MRI images (**c, d**). The normal right ear (**c**) demonstrated ^23^Na MRI grade 3 signal lateral to the internal auditory meatus (vertical open arrow) but the anatomical compatibility of this signal was uncertain and scored as grade 1 (minor overlap). However, following registration and fusion (**c**) there was noted to be major overlap with the inner ear structures. The pathological EH left ear (**b** and **d**) did not demonstrate any visible ^23^Na MRI inner ear signal (grade 0) lateral to the internal auditory meatus (vertical open arrow). Note the ^23^Na MRI signal from the inferior temporal lobes which lies anterior to the cochlea on each side. The ^23^Na MRI images grey scale imaging may be accessed in the supplementary data

The two observers independently analysed the ^23^Na MR images in isolation and then with the ^23^Na MR images registered and fused to the T2w SPACE sequence (Figs. 2,3), whilst blinded to clinical and delayed post-GBCA 3D-IR data. The visibility and anatomical compatibility of ^23^Na MRI signal in each IAM, cochlea and vestibule were qualitatively graded with Likert scales (Table 2). The visibility grades were based on the qualitative evaluation of ^23^Na MR signal intensity (NaSI) when compared to that returned by adjacent structures (Table 2). The grading of anatomic compatibility was based on prior anatomical knowledge for the ^23^Na MR images in isolation and depended on overlap with the corresponding anatomy on the T2w SPACE sequence for the registered and fused images (Table 2). The certainty of laterality was classified by comparing the difference between cochlea or vestibule visibility grade and the adjacent fundus of the IAM between the two sides. If either cochlea or vestibule visibility grade (relative to the fundus of the IAM) was 1 point greater than the contralateral ear and it had least grade 2 anatomic compatibility on both sides, then it was classified as possible laterality. If either cochlea or vestibule visibility grade was 2 points greater (relative to the fundus of the IAM) than the contralateral ear and it had at least grade 2 anatomic compatibility on both sides, then it was classified as definite laterality.

### Segmentation and quantitative analysis

**Fig. 4:**
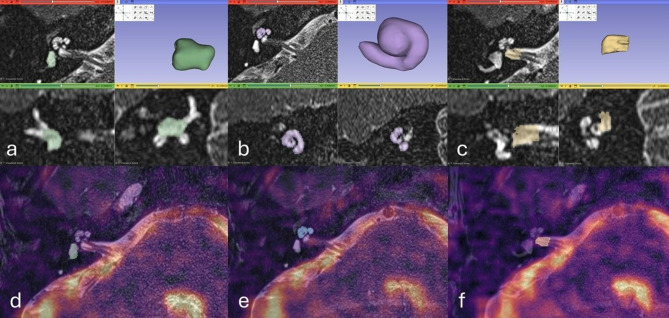
Screenshots from 3D-Slicer 5.6.2 illustrate the segmentation of the inner ear structures and the internal auditory meatus. Segmentations of the (**a**) vestibule, (**b**) cochlea and (**c**) lateral internal auditory meatus are shown with multiplanar reformatted images and 3D-rendering (top right in each pane). The corresponding segmentations of the (**d**) vestibule, (**e**) cochlea and (**f**) lateral internal auditory meatus are overlaid on axial fused registered ^23^Na MRI- T2-SPACE images

The observers subsequently manually segmented each cochlea, vestibule and lateral half of IAM on the T2w SPACE images, facilitated by comparable intensity-based thresholding (Fig. 4). The manually segmented masks were visualized by 3D maximum intensity projections to ease quality assessment. The segmentations were then transferred to the transformed ^23^Na MRI, and the SI statistics were extracted for each of the segmented volumes.

### Statistical analysis

The mean, standard deviation and median of the NaSI for each cochlea and vestibule was documented, and the ratio of the median cochlea and vestibule NaSI to that of the adjacent IAM was calculated. Intraclass correlation coefficients (ICC) evaluated the absolute agreement of inner ear (cochlea and vestibule) median NaSI between the two observers. The small sample size precluded reliability statistics for the qualitative analysis [26].

## Results

### Participant characteristics

There were 4 eligible participants imaged (age mean 60.3 years, range 33–78 years; 2f, 2 m) with a mean duration of symptoms of 16 years (range 3–40 years). The laterality of MD, audio-vestibular symptoms, audiogram and vestibular function tests are presented in Table 3. Complete replacement of the vestibular perilymph by the dilated endolymphatic structures was evident on delayed post-GBCA MRI in 3/4 participants.

**Table 3:** Demographic, clinical and delayed post-gadolinium MRI data for the four participants

Participant	Age/sex	Ménière’s Disease laterality	Symptoms	Vestibular function tests	**Duration of symptoms/Interval from most recent vertigo episode to research** ^**23**^**Na MRI**	Audiogram	**Time interval between clinical MRI and research** ^**23**^**Na MRI**	Subsequent Ménière’s Disease activity at follow up	Nakashima hydrops MRI grade (19) ipsilateral cochlea/ vestibule	Nakashima hydrops MRI grade (19) contralateral cochlea/ vestibule
A	78/M	left	Ipsilateral tinnitus, HL, vertigo (3–5 hours)	Ipsilateral calorics/VNG abnormal Contralateral normal	17 years/ 1 month	Ipsilateral pan-frequency Contralateral panfrequency	60 months	No vertigo attacks at 12 months follow up	2/2 Complete replacement of the vestibule by endolymph	0/0
B	62/F	right	Ipsilateral tinnitus, HL, vertigo (2 min-1 hour)	Ipsilateral calorics/ VHIT/VNG abnormal Contralateral normal	36 months/ 30 months	Ipsilateral pan-frequency Contralateral HFSNHL > 3K	31 months	No follow up	2/2 Complete replacement of the vestibule by endolymph	0/0
C	68/M	left	Ipsilateral tinnitus, HL, aural fullness, vertigo (30 min-12 hours)	Ipsilateral VHIT/VNG normal	40 years/ 3 months	Ipsilateral pan-frequency Contralateral HFSNHL > 6K	7 months	Two vertigo attacks at 20 months follow up	2/2 Complete replacement of the vestibule by endolymph	0/0
D	33/F	left	Ipsilateral tinnitus, HL, aural fullness, vertigo (3–4 hours)	None performed	48 months/ 12 months	Ipsilateral pan-frequency Contralateral normal	9 months	No vertigo attacks at 18 months follow up	2/2 > 50% replacement of the vestibule by endolymph	0/0

**HL** Hearing loss; **HFSNHL** High frequency sensorineural hearing loss; **VHIT** Video head impulse test; **VNG** Videonystagmograph

### Qualitative analysis

The outcomes of the qualitative analysis for image quality, IAM and inner ear visibility, anatomic compatibility and laterality are tabulated (Table 4) and illustrated in Figs. 2 and 3 (normal right ears and hydropic left ears). Eight ears in four participants were analysed by two observers (total of 16 observations). ^23^Na MRI image quality was suboptimal with at least a moderate effect of artefact in 2/4 studies, largely due to distortion, blurring and ghosting resulting from movement artefact. The optimal frequency offsets selected for the depiction of each inner ear were (right/left) +200 Hz/+200 Hz, +40 Hz/-60 Hz, −60 Hz/-160 Hz and +60 Hz/-100 Hz for participants A, B, C and D respectively. The IAMs were at least partially visible in all ^23^Na MRI studies prior to registration and fusion, with anatomical compatibility grades of 2 or 3 indicating that the majority of the IAM was depicted in 10/16 observations. When considering the cochlea and vestibule separately, following registration and fusion of ^23^Na MRI, anatomical compatibility grades increased in 6/32 observations, decreased grades in 4/32, and remained the same in 22/32. Grade 2 or 3 anatomical compatibility grades were achieved in 12/16 normal inner ear observations following registration and fusion.

**Table 4:** Qualitative analysis of ^23^Na MRI studies for the four participants: image quality, artefact, signal visibility, anatomical compatibility and lateralisation

Participant	Image quality	Artefact		Observer	Signal visibility/Anatomical compatibility						*Certainty of hydrops laterality and side	Side of laterality correct/incorrect
					Right IAM	Right cochlea	Right vestibule	Left IAM	Left cochlea	Left vestibule		
A	1	1	Pre-registration	1	1/1	3/1	3/1	1/2	**0/0**	**1/1**	None	NA
				2	1/2	3/2	3/2	1/2	**0/0**	**0/0**	Definite/left	**Correct**
			Post-registration	1	1/1	3/2	3/2	1/2	**0/0**	**1/1**	Definite/left	**Correct**
				2	1/2	3/2	3/2	1/2	**0/0**	**0/0**	Definite/left	**Correct**
B	2	2	Pre-registration	1	2/2	**2/1**	**2/1**	2/3	1/1	1/1	None	NA
				2	1/1	**1/1**	**1/1**	2/2	1/1	1/1	None	NA
			Post-registration	1	1/2	**2/1**	**2/1**	2/2	1/1	1/1	None	NA
				2	1/1	**1/1**	**0/0**	2/3	1/2	1/2	None	NA
C	2	2	Pre-registration	1	1/3	1/3	1/3	1/3	**1/1**	**0/0**	Possible/left	**Correct**
				2	1/3	1/2	1/2	1/3	**0/0**	**1/1**	Possible/left	**Correct**
			Post-registration	1	1/3	1/3	1/3	1/3	**0/0**	**0/0**	Possible/left	**Correct**
				2	1/3	1/2	1/2	1/3	**0/0**	**0/0**	Possible/left	**Correct**
D	1	0	Pre-registration	1	1/1	1/1	1/1	1/1	**0/0**	**0/0**	None	NA
				2	1/1	2/1	2/1	1/1	**1/1**	**1/1**	None	NA
			Post-registration	1	I/2	2/2	1/1	1/1	**0/0**	**0/0**	Definite/left	**Correct**
				2	1/1	2/1	2/2	1/1	**0/0**	**1/1**	Possible/left	**Correct**

IAM; internal auditory meatus

Inner ear grades in the inner ear with EH are in **bold**

*The certainty of laterality was based on the relative difference in visibility of either the cochlea or vestibule to the IAM fundus when comparing the two sides. If either cochlea or vestibule visibility grade (relative to the fundus of the IAM) was 1 point greater than the contralateral ear and it had least grade 2 anatomic compatibility, then it was classified as possible laterality. If either cochlea or vestibule visibility grade was 2 points greater (relative to the fundus of the IAM) than the contralateral ear and it had at least grade 2 anatomic compatibility, then it was classified as definite laterality

Prior to registration and fusion, the anatomical compatibility and visibility grades indicated EH lateralisation in only 1/4 participants (participant C) for both observers. However, following registration and fusion with structural T2w SPACE imaging, there were 3/4 participants with lateralisation correctly independently predicted by both observers.

### Quantitative analysis

The mean, standard deviation and median inner ear NaSI are shown for the cochlea (Table 5) and the vestibule (Table 6). The mean (range) of cochlea volume was 83 (63–110) mm^3^ for observer 1 and 80 (59–115) mm^3^ for observer 2 (ICC 0.80), whilst the mean (range) of vestibular volume was 47 (31–59) mm^3^ for observer 1 and 41 (33–50) mm^3^ for observer 2 (ICC 0.51).

**Table 5:** Quantitative analysis of the cochlea on ^23^Na MRI studies for the four participants

Participant		Endolymphatic hydrops **cochlea signal intensity (x10**^**−5**^)				Normal **cochlea signal intensity (x10**^**−5**^)				*Ratio of normal cochlea median signal: hydropic cochlea median signal	*Ratio of normal cochlea/IAM median signal: hydropic cochlea/IAM median signal
		Mean	SD	Median	Median cochlea/Median IAM signal	Mean	SD	Median	Median cochlea/ Median IAM signal		
A	Observer 1	6.4	2.7	6.2	0.7	8.4	3.8	7.9	1.7	1.5:1	2.0:1
	Observer 2	5.4	2.1	6.0	0.6	10.1	4.5	10.1	1.4		
B	Observer 1	7.0	1.3	7.7	0.9	5.5	2.9	6.4	0.8	0.8:1	0.8:1
	Observer 2	6.0	3.2	7.1	1.1	5.7	2.6	5.9	0.7		
C	Observer 1	5.5	2.5	4.7	0.4	4.9	1.1	5.1	0.5	1.2:1	1.2:1
	Observer 2	4.8	2.8	3.6	0.4	3.9	1.9	4.5	0.5		
D	Observer 1	6.3	3.0	5.2	0.6	5.9	3.9	8.2	1.7	1.3:1	2.8:1
	Observer 2	3.5	1.5	3.5	0.3	4.3	3.1	2.9	0.5		

Calculated from the mean of the two observers

**Table 6:** Quantitative analysis of the vestibule on ^23^Na MRI studies for the four participants

Participant		Endolymphatic hydrops **vestibule signal intensity (x10**^**−5**^)				Normal **vestibule signal intensity (x10**^**−5**^)				*Ratio of Normal vestibule median signal: hydropic vestibule median signal	*Ratio of normal vestibule/IAM median signal: hydropic vestibule/IAM median signal
		Mean	SD	Median	Median vestibule/Median IAM signal	Mean	SD	Median	Median vestibule/ Median IAM signal		
A	Observer 1	6.3	3.3	5.7	0.6	9.1	4.8	10.3	1.3	2.1:1	2.9:1
	Observer 2	4.6	1.1	4.6	0.4	9.5	5.0	11.7	1.6		
B	Observer 1	6.3	2.4	7.1	0.8	5.6	2.7	5.9	0.8	0.8:1	0.7:1
	Observer 2	9.5	5.0	9.7	1.3	6.0	2.3	7.0	0.8		
C	Observer 1	4.4	2.3	3.2	0.3	6.0	3.3	7.9	0.7	1.9:1	1.9:1
	Observer 2	4.1	1.7	3.3	0.4	5.2	2.4	4.3	0.5		
D	Observer 1	6.5	4.4	6.9	0.8	5.6	3.0	5.1	1.1	1.1:1	2.2:1
	Observer 2	4.6	3.0	4.3	0.4	5.8	3.7	7.7	1.4		

Calculated from the mean of the two observers

Based on the average ratings from the two observers, the normal: EH median NaSI ratios for the cochlea were 1.4:1, 0.8:1, 1.2:1, and 1.3:1 in the four participants. For the vestibule, the normal: EH median NaSI ratios were 2.1:1, 0.8:1, 1.9:1, and 1.1:1. The median NaSI was decreased in both the cochlea and vestibule of the EH ears in the 3/4 participants (participants A, C and D) who had the correct lateralisation on qualitative analysis following registration and fusion. When the median inner ear NaSI was scaled to the adjacent IAM NaSI, the corresponding ratios were 2.0:1, 0.8:1, 1.2: 1 and 2.8: 1 (mean 1.7:1) for the cochlea and 2.9:1, 0.7:1, 1.9:1 and 2.2: 1 (mean 1.9:1) for the vestibule. The ICC for the inner ear median NaSI was 0.70 (95% CI; 0.15–0.89).

## Discussion

A ^23^Na MRI bSSFP sequence was developed for the demonstration of the inner ears on a 7T MRI system. In four participants with unilateral MD, both observers correctly lateralised the ear with severe vestibulo-cochlear endolymphatic hydrops in only 1/4 participants with a signature of reduced inner ear signal on qualitative assessment of ^23^Na MRI data alone. However, when ^23^Na MRI was registered and fused to a T2 SPACE sequence, there was successful endolymphatic hydrops lateralisation by both observers in 3/4 participants. There was no incorrect lateralisation of EH by either observer either before or after registration and fusion. It was not possible to lateralise in participant B, and the potential reasons for this are discussed below. On quantitative analysis of segmented inner ear structures, the same 3/4 participants demonstrated increased ^23^Na MRI median signal intensity in both the cochlea (1.2–1.5 times) and vestibule (1.1–2.1 times) in the normal ear relative to the symptomatic MD ear. The ratio between the median signal intensity of the normal and the pathological inner ears increased when it was scaled to the signal intensity of the adjacent IAM, with a 1.2–2.8:1 ratio for the cochlea and a 1.9–2.9:1 ratio for the vestibule.

There are no previous reports of ^23^Na MRI being applied to inner ear imaging, with considerable challenges resulting from the small volume anatomical structures and the variation in static magnetic field (B_0_) due to juxtaposition of bone, air and fluid. Our ^23^Na MRI research sequence and post-processing was adapted to maximise SNR efficiency at adequate spatial resolution and to address artefacts due to off-resonance frequencies. Acquisition with a fast (non-Cartesian) 3D-Cones trajectory was employed to reduce the duration of signal readout, as required by the rapid T2* decay of ^23^Na, and to improve SNR [18]. A bSSFP sequence was also utilised to boost SNR [19], with the banding artefact minimised by utilising a short TR and employing phase-cycling [20] with signal averaging across cycles. It was decided a priori that a 30-minute sequence would be reasonable from the patient perspective, but 2/4 of the MRI studies demonstrated suboptimal image quality due to participant movement. More targeted array coils with parallel imaging strategies [27] and methods to reconstruct high-quality MRI data from limited k-space data [28] may optimise 7T ^23^Na MRI inner ear imaging in the future.

The findings of this study indicate that ^23^Na MRI can detect changes in MD, inviting exploration of other potential applications in inner ear pathologies. Firstly, ^23^Na MRI may have a role in evaluating interval changes in size of the endolymphatic compartment and monitoring the evolution of EH, since structural imaging has been proven to be limited in its ability to demonstrate treatment response and explain fluctuations in symptoms [29, 30]. Secondly, there may be a wider role for ^23^Na MRI in probing the ionic milieu of the inner ear as an insight to disease processes. It is recognised that increases or variations in endolymph sodium concentrations result in cellular dysfunction, and clinical audio-vestibular disorders [31, 32]. However, there are logistical and technical difficulties in obtaining fluid samples in vivo, since it is both invasive and the composition may be disturbed during surgical procedures [33]. ^23^Na MRI could offer a non-invasive bio-marker to evaluate the sodium concentration of the pathological inner ear in the presence of normal structural MRI appearances. For instance, abnormalities of sodium haemostasis have been linked with other inner ear conditions including sudden onset hearing loss due to ischaemic anoxia [34–36] and genetic conditions such as non-syndromic autosomal recessive deafness (DFNA8/10) [37]. Thirdly, there may also be a role in assessing the impact of systemic alterations of sodium concentration on inner ear metabolism [38]. In this regard, it has long been observed that otologic symptoms are aggravated after high salt intake [39, 40] whilst low sodium intake is beneficial in MD [41]. Finally, it should be considered whether targeting the evaluation to individual inner ear structures may be beneficial. Since EH is able to replace a greater proportion of the vestibular PS than is possible in the cochlea, this may be the focus of future studies in MD, and our data did confirm a greater degree of vestibular than cochlear NaSI asymmetry.

In participant B, there was no qualitative lateralisation of EH or increased ^23^Na MRI median signal intensity in the normal ear relative to the MD ear, despite good image quality. This leads us to re-examine the assumption in our hypothesis, which was that the hydropic ES would always demonstrate low ^23^Na concentrations like the normal ES. There is some data to suggest that there may be paradoxical increases in the ^23^Na concentrations in the presence of EH [42–45], which would diminish NaSI asymmetry and may explain the findings in this participant. Potential mechanisms include the reduced extraction of ^23^Na from the endolymph in the extra-osseous endolymphatic sac [13, 43, 44] or direct communications between the ES and PS [45]. Changes in endolymphatic ^23^Na concentrations have also been implicated in premenstrual exacerbation [46] and genetic forms [47] of MD, whilst animal studies of EH have also shown 2-3x increases in ES ^23^Na concentration [48]. It is of interest that participant B had the longest interval between a prior vertiginous episode and the ^23^Na MRI study and a hypothesis that this might be a feature of inactive disease should be investigated in future studies.

There are study limitations which should be documented. Firstly, the exploratory nature of the study should be emphasised, with the sample size being limited in its ability to allow statistical analysis and firm conclusions. Assessing the clinical validity of ^23^Na MRI will require a larger cohort of patients with MD and a normal control population. Secondly, the aforementioned technical challenges in obtaining adequate inner ear SNR limited the spatial resolution, with a nominal resolution of 2 mm but with an estimated full width at half maximum of the point spread function estimated to be 2.88 mm. The inner ear segmentation volumes were concordant with previous estimates [49] such that the vestibule corresponded to a maximum of 5 voxels and the cochlea to a maximum of 10 voxels on ^23^Na MRI. This limited the confidence of registration and accurate quantification of inner ear signal. Thirdly, methodological aspects should be addressed. The selection of the optimal frequency offset was subjective and non-standardised so would benefit from automation in the future. The landmark-based registration process was also user dependent and potentially impacted on the reliability of quantitative measures. Automated registration or emerging deep-learning based prediction of the transform maybe a more robust approach for future studies. An alternative method would be to acquire spatially aligned proton MRI structural imaging concurrently with ^23^Na MRI to aid registration, but this was precluded in our study by the lack of a receiver array for proton in the dual tuned coil, which precludes acquisition of inner ear images of the required resolution. It should also be considered that 7–60 months had elapsed between the clinical and the research MRI studies with the potential for interval changes. Previous longitudinal studies have indicated the possibility of both EH progression in the asymptomatic ear as well as variable EH changes in the symptomatic ear which may have influenced the outcomes [50–52]. Whilst the contralateral MD ear may be considered a suboptimal control in view of the potential to develop bilateral MD, this was mitigated by the selection of patients without any evidence of EH in the contralateral ear on delayed post-gadolinium 3D-IR MRI. However, it may be argued that there is the potential for metabolic derangement in the contralateral MD ear without the manifestation of EH, so alternative control groups should be considered in future studies. Finally, we should be guarded about the wider applicability of these methods and results. Only patients with unilateral severe EH were examined, so the results may not be generalised to milder forms. In addition, the evaluation of asymmetry would not be pertinent to bilateral MD. Whilst the potential to apply absolute inner ear NaSI in this setting would warrant further investigation, the overlap between the NaSI values of pathological EH and normal ears in this study would argue against this approach. Moreover, the relevance to routine clinical scanning is limited since ^23^Na coils and ultra-high-field MRI are not currently in widespread use. In this regard, translation to lower field strength using iterative denoising reconstruction algorithms should be investigated.

## Conclusion

This exploratory study demonstrates the potential for qualitative analysis of 7T ^23^Na MRI to lateralise severely hydropic ears in patients with unilateral definite MD, although there remain technical challenges to acquisition and post-processing. Optimal evaluation required registration and fusion with structural imaging and was supported by the results of quantitative analysis. The absence of qualitative and quantitative NaSI asymmetry in one participant, should lead to caution in accepting the clinical validity of this technique and may challenge the underlying assumption that there are low sodium concentrations in the hydropic ES. Despite the limitations, this study provides a proof of principle for the potential role of ^23^Na MRI in the evaluation of MD without the requirement for gadolinium-based contrast agents and supports a theoretical basis for its wider evaluation in MD and other inner ear pathologies.

## Data Availability

The data supporting this article has been deposited in the King’s College London research data repository, KORDS, at 10.18742/28368968. It is not openly available due to conditions of participant consent and may be shared with researchers on request.

## Abbreviations

Bssfp: Balanced steady-state free precession
3D-FLAIR: 3D Fluid Attenuated Inversion Recovery
3D-IR: Three-dimensional inversion recovery
EH: Endolymphatic hydrops
ES: Endolymphatic space
FLASH: Fast low angle shot
GBCA: Gadolinium-based contrast agents
IR: Inversion recovery
MD: Ménière’s disease
MRI: Magnetic resonance imaging (MRI).
PS: Perilymphatic space
^23^Na: Sodium
NaSI: ^23^Na sodium MRI signal intensity
SAR: Specific absorption rate
SI: Signal intensity
SNR: Signal to noise ratio
SPACE: Sampling perfection with application optimised contrasts using different flip angle evolution
T: Tesla
T2w: T2 weighted
TR: Repeat time
